# A comparison of methods for estimating substitution rates from ancient DNA sequence data

**DOI:** 10.1186/s12862-018-1192-3

**Published:** 2018-05-16

**Authors:** K. Jun Tong, David A. Duchêne, Sebastián Duchêne, Jemma L. Geoghegan, Simon Y. W. Ho

**Affiliations:** 1 School of Life and Environmental Sciences, University of Sydney, Sydney, Australia; 2 Department of Biochemistry and Molecular Biology, Bio21 Molecular Science and Biotechnology Institute, University of Melbourne, Melbourne, Australia; 3 Department of Biological Sciences, Macquarie University, Sydney, Australia

**Keywords:** Ancient DNA, Tip dating, Least-squares dating, Bayesian phylogenetics, Substitution rate, Mitogenomes

## Abstract

**Background:**

Phylogenetic analysis of DNA from modern and ancient samples allows the reconstruction of important demographic and evolutionary processes. A critical component of these analyses is the estimation of evolutionary rates, which can be calibrated using information about the ages of the samples. However, the reliability of these rate estimates can be negatively affected by among-lineage rate variation and non-random sampling. Using a simulation study, we compared the performance of three phylogenetic methods for inferring evolutionary rates from time-structured data sets: regression of root-to-tip distances, least-squares dating, and Bayesian inference. We also applied these three methods to time-structured mitogenomic data sets from six vertebrate species.

**Results:**

Our results from 12 simulation scenarios show that the three methods produce reliable estimates when the substitution rate is high, rate variation is low, and samples of similar ages are not all grouped together in the tree (i.e., low phylo-temporal clustering). The interaction of these factors is particularly important for least-squares dating and Bayesian estimation of evolutionary rates. The three estimation methods produced consistent estimates of rates across most of the six mitogenomic data sets, with sequence data from horses being an exception.

**Conclusions:**

We recommend that phylogenetic studies of ancient DNA sequences should use multiple methods of inference and test for the presence of temporal signal, among-lineage rate variation, and phylo-temporal clustering in the data.

**Electronic supplementary material:**

The online version of this article (10.1186/s12862-018-1192-3) contains supplementary material, which is available to authorized users.

## Background

Estimating the rate of molecular evolution is a key step in inferring evolutionary timescales and population dynamics from genetic data. In turn, these estimates can provide useful insights into various biological and population processes [1]. Evolutionary rates can be inferred using phylogenetic methods based on molecular clocks, provided that they can be calibrated using independent information about time. When genetic data sets are time-structured, with samples having been drawn at distinct points in time, the ages of the DNA sequences themselves can be used for calibration [2, 3].

Time-structured sequence data are common in studies of rapidly evolving genomes, such as those of pathogens [4]. They can also be obtained by sequencing DNA from ancient samples of animals, plants, and fungi [5]. In these cases, the sample ages can be inferred by radiometric dating or stratigraphic correlation. When relying on the tip dates for calibration, an important condition is that the population must be ‘measurably evolving’ [6], whereby the sampling window is wide enough to capture an appreciable amount of genetic change. Importantly, this depends on the evolutionary rate, which varies across genes and species [7, 8]. Assembling data sets with sufficient temporal structure can be difficult to achieve for slowly evolving organisms such as vertebrates [9, 10].

There are several methods for estimating substitution rates from time-structured sequence data [10]. The simplest approach is based on linear regression of root-to-tip (RTT) distances, taken from an estimated phylogram, against the ages of the corresponding sequences [11, 12]. RTT regression is based on the expectation that, from the time of the most recent common ancestor, ancient sequences have accumulated less genetic change than their younger counterparts. Assuming that molecular evolution has been clocklike, the slope of the regression line provides an estimate of the substitution rate. A key drawback of this method is that the data points are not phylogenetically independent, because some of the branches in the tree contribute to multiple root-to-tip measurements [6, 11].

Least-squares dating is another computationally efficient method for inferring rates from time-structured data [13]. It assumes a strict clock and fits a curve to the data using a normal approximation of the Langley-Fitch algorithm [14]. This approximation is somewhat robust to departures from rate homogeneity among lineages. Least-squares dating and RTT regression both require a fixed tree topology and cannot directly take into account or report phylogenetic uncertainty. These two methods are commonly used in analyses of rapidly evolving pathogens, but have rarely been applied in studies of ancient DNA from eukaryotes.

Bayesian phylogenetic methods can be used for joint estimation of substitution rates and the tree [15], allowing the estimate of the rate to be marginalized over the uncertainty in the tree topology and other model parameters. These methods have a number of advantages: they can account for phylogenetic uncertainty, allow the error in sequence ages to be specified [16, 17], and enable the joint estimation of other evolutionary and demographic parameters of interest [15, 18]. Moreover, the use of relaxed-clock models allows rate variation across branches to be taken into account [19]. In studies of ancient DNA, Bayesian phylogenetic methods also allow post-mortem decay to be modelled, as either an age-dependent [20] or age-independent process [21].

Analyses of ancient DNA data have typically yielded very high rate estimates compared with those obtained using fossil-based calibrations at internal nodes [9, 22–24], partly because they capture short-term evolutionary dynamics that involve features such as incomplete purifying selection [25]. However, rate estimates from time-structured sequence data are subject to several potential sources of error [10]. Biases can be caused by tree imbalance [26], closely related samples having the same age (phylo-temporal clustering) [27, 28], the presence of strong population structure [29], and rate variation among lineages [30]. The relative impacts of these factors and their behaviour across commonly used methods of inference remain poorly understood.

In a recent study of 81 data sets from viruses, relatively congruent estimates of substitution rates were obtained using RTT regression, least-squares dating, and Bayesian phylogenetic analysis [31]. High among-lineage rate variation was the only feature of the data to be significantly associated with incongruence across the rate estimates from different methods. However, phylo-temporal clustering also tended to be greater in data sets that yielded different rate estimates across the three estimation methods [31].

Ancient DNA sequences from eukaryotes present different analytical challenges compared with serial samples from viruses [6]. For instance, ancient DNA sequences are often difficult to obtain, so that they are typically sampled from a limited number of time points. In studies of extant species, ancient DNA sequences are usually outweighed by sequence data from modern samples. In addition, substitution rates are generally lower in eukaryotes than in bacteria and viruses [4], and the sampling window of the sequences often represents only a small fraction of the time to their most recent common ancestor [32]. Collectively, these characteristics mean that ancient DNA data sets from eukaryotes are more likely to lack sufficient temporal structure for reliable inference of substitution rates [9]. The impacts of these features of ancient DNA data potentially vary across different methods of rate estimation.

In this study, we use simulations to examine two potential sources of error in rate estimates from ancient DNA data: complex patterns of rate heterogeneity among lineages, and sampling schemes with phylo-temporal clustering. We investigate the impacts of these factors on rate estimates made using RTT regression, least-squares dating, and Bayesian phylogenetic analysis. We also compare the rate estimates from these three methods in analyses of time-structured mitogenomic data sets from six vertebrate species.

## Methods

### Simulations

We simulated genealogies of 100 tips in BEAST 2 [33], by sampling from a constant-size coalescent prior conditioned on the ages of the tips. In all cases, the age of the root was fixed to 500,000 years and half of the tips corresponded to present-day samples. The ages of the other 50 tips were randomly distributed between the present and 50,000 years ago (i.e. 10% of the age of the root). These conditions were chosen to resemble those of typical ancient mitogenomic data sets from vertebrates [34, 35] (Additional file 1: Figure S1). Trees had two different degrees of phylo-temporal clustering, with 100 replicates each: high clustering was simulated by making all present-day samples form a monophyletic group, whereas low clustering was simulated by only making half of the present-day samples form a monophyletic group.

Using the simulated genealogies and the program NELSI [36], we varied the mean substitution rate and the degree of among-lineage rate variation. Simulations were performed using two substitution rates that span the range of rates in most molecular dating studies of ancient mitogenomes [23, 37]: a high rate of 10^− 7^ subs/site/year and low rate of 10^− 8^ subs/site/year. For each of the two rate schemes, we simulated three scenarios of among-lineage rate variation under a white-noise model [38], with variance along each branch of 0.1% (low), 1% (medium), and 10% (high) of the expected number of substitutions. Sequence evolution was simulated according to the HKY + Γ substitution model using the R package phangorn [39] for each of the 100 tree replicates in the 12 different scenarios. All sequences had lengths of 15,000 nucleotides, to reflect the approximate size of vertebrate mitogenomes. Our trees and sequence alignments are available for download from Github (github.com/kjuntong/aDNA_Rates_BMCEvoBio).

For the 100 data sets in each simulation treatment, we used three methods to estimate the substitution rate. The first was RTT regression in TempEst 1.5 [11]. The second method was least-squares fitting in LSD 0.3 [13], with the ages of the samples used to constrain the least-squares fitting algorithm. These two methods require an estimated tree with branch lengths in substitutions per site; we inferred the topology and branch lengths using maximum likelihood and the HKY + Γ substitution model in RAxML v8.2.4 [40]. In each case, a rapid bootstrapping analysis with 100 replicates was followed by a search for the best-scoring tree. The bootstrap replicates were only used to provide starting points when searching for the best-scoring tree, but not to measure node support for the inferred trees.

The third method that we used to analyse the data was Bayesian phylogenetic inference in BEAST 1.8.3 [41]. We used an uncorrelated lognormal relaxed clock [19], constant-size coalescent tree prior, and HKY + Γ substitution model. As an uninformative prior on the mean substitution rate, we used the conditional reference prior described by Ferreira and Suchard [42]. Posterior distributions of parameters were estimated by Markov chain Monte Carlo (MCMC) sampling. Samples were drawn every 5000 steps over a total of 50 million steps, with the first 10% of samples discarded as burn-in. We considered that sampling was sufficient when the effective sample size of every parameter exceeded 200, as estimated using LogAnalyser in the BEAST package. Where required, we ran additional MCMC analyses to achieve sufficient sampling.

To examine the differences in the accuracy of rate estimates for data sets generated under the various simulation treatments, we calculated the standardized error in rate estimates for each simulation as the difference between the estimated and true rates, divided by the true rate. We used one-sample Wilcoxon tests to evaluate whether the distribution of standardized errors from each scenario of simulation and estimation was different from zero. Standardized errors were also compared between scenarios using a Kruskal-Wallis one-way analysis of variance, and post-hoc pairwise Mann-Whitney-Wilcoxon tests.

### Mitochondrial genomes

We obtained a range of time-structured mitogenomic data sets from previous studies and from GenBank (Table 1). These included complete sequences of mitochondrial genomes from the Adélie penguin (*Pygoscelis adeliae*) [23], brown bear (*Ursus arctos*) [43], domestic dog (*Canis familiaris*) [44], horse (*Equus caballus*) [45–47], modern human (*Homo sapiens*) [48], and woolly mammoth (*Mammuthus primigenius*) [49]. The sampling windows of these data sets ranged from 7134 to 122,500 years and the number of sequences in each data set ranged from 20 to 237 (Table 1). We partitioned each data set into five subsets: first codon positions of protein-coding genes, second codon positions, third codon positions, control region, and rRNA genes. Our mitogenomic data sets, including the subsets of the data, are available on Github (github.com/kjuntong/aDNA_Rates_BMCEvoBio).

**Table 1:** Six time-structured mitogenomic data sets analysed in this study

Species	Scientific name	Tips (modern + ancient)	Length (nt)	Age range (years before present)	Outgroup^a^	Main sources^b^
Adélie penguin	*Pygoscelis adeliae*	13 + 7	14,198	0–44,000	*Pygoscelis antarctica*	[23]
Brown/polar bear	*Ursus arctos* & *U. maritimus*	31 + 1	14,609	0–122,500	*Ursus americanus*	[43]
Dog	*Canis familiaris*	120 + 18	14,596	0–36,000	*Canis latrans*	[44]
Horse	*Equus caballus*	147 + 20	14,910	0–42,577	*Equus asinus*	[45–47]
Modern human	*Homo sapiens*	200 + 37	14,893	0–7134	*Homo neanderthalensis*	[48]
Woolly mammoth	*Mammuthus primigenius*	0 + 65	14,951	12,210–46,455	*Elephas maximus*	[49]

^a^GenBank accession numbers for outgroup sequences are given in the sequence data files

^b^Main publications from which the sequence data were obtained

For each mitogenomic data set, we estimated the substitution rate with the same three methods that were compared in our simulation study. In all analyses, the sampling times of the sequences were used for calibration; no age constraints were applied to internal nodes in the tree. To infer phylograms for TempEst and LSD, we used maximum likelihood in RAxML with an HKY + Γ model of nucleotide substitution for each data subset. In each case, a rapid bootstrapping analysis with 100 replicates was followed by a search for the best-scoring tree. Outgroup sequences were included in order to allow the position of the root to be estimated in the phylograms (Table 1), but were pruned from the tree for subsequent analyses of substitution rates.

We performed Bayesian phylogenetic analysis of each data set using BEAST 1.8.3. A separate HKY + Γ model of nucleotide substitution was assigned to each subset of the mitogenome data. We used a continuous-time Markov chain reference prior for the substitution rate [42], with each subset of the data allowed a distinct relative rate. Posterior distributions of parameters were estimated by sampling every 5000 steps over a total of 50 million MCMC steps. We ran each analysis in duplicate to check for convergence, and the samples from the two runs were combined after discarding the first 10% of samples as burn-in. Sampling was considered to be sufficient when the effective sample size of each parameter exceeded 200.

In order to compare the fit of different coalescent tree priors (constant-size and skyride [50]) and clock models (strict clock and uncorrelated lognormal relaxed clock [19]), we computed marginal likelihoods using stepping-stone sampling [51]. The combination of tree prior and clock model that yielded the highest marginal likelihood was considered to be the best fitting. However, we preferred the simpler tree prior or clock model when its marginal likelihood was within 1 log unit of the more parameter-rich alternative. This is consistent with the guidelines offered by Kass and Raftery [52], who proposed that a difference of 1 log unit in marginal likelihoods is required to constitute positive evidence for one model over another.

We checked each data set for temporal structure using a date-randomization test [53]. In this test, the sample ages are randomly reassigned to the sequences and the analysis of the data is repeated. This is done a number of times in order to generate a set of rate estimates from date-randomized data sets. To evaluate the temporal structure in the data, we considered two criteria that have been proposed for the date-randomization test, CR1 and CR2 [27]. Under CR1, the rate estimate from the original data set should not be included within the 95% credibility intervals of the rate estimates from the date-randomized replicates. Under the stricter criterion CR2, the 95% credibility interval of the rate estimate from the original data set should not overlap with the 95% credibility intervals of the rate estimates from the date-randomized replicates. Our results are based on 20 date-randomized replicates of the original data.

For each data set, we evaluated the degree of phylo-temporal clustering by correlating the distances between tips in the tree with the ages of those tips [31]. For each pair of tips, we measured distance as the number of nodes separating them, and took the difference in their sampling times. We then calculated the Pearson’s correlation coefficient (*ρ*) for the entire data set. We calculated a *P*-value by generating a null distribution of *ρ* by randomizing the sampling times in the trees 1000 times. A significant association between sampling times and phylogenetic distance is indicated by *P* < 0.05.

## Results

### Simulations

**Fig. 1:**
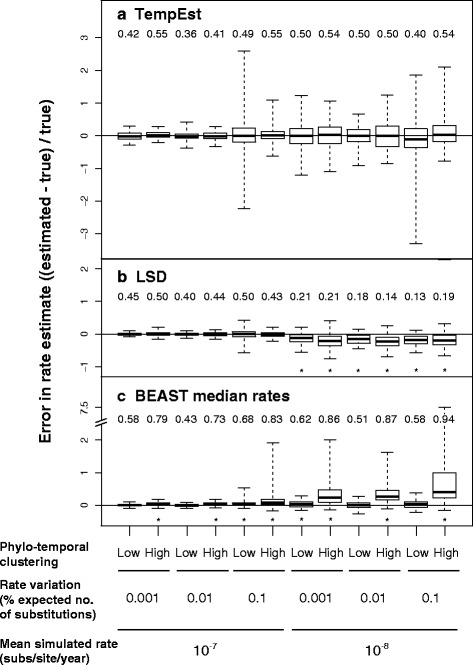
Standardized error in estimates of substitution rates from sequence data produced under 12 different simulation conditions, representing different combinations of substitution rate, rate variation among lineages, and phylo-temporal clustering. Data were analysed using **a** regression of root-to-tip distances in TempEst, **b** least-squares dating in LSD, and **c** Bayesian inference in BEAST. Numbers along the top of each panel indicate the proportion of estimates that are above the rate used for simulation (‘true’ rate). Asterisks indicate cases in which one-sample Wilcoxon tests of errors in rate estimates show a significant difference from zero (see Additional file 2: Table S1)

The three methods of rate estimation, RTT regression, least-squares fitting, and Bayesian estimation, produced more accurate rate estimates for sequence data produced by simulation using a high rate than using a low rate (Fig. 1; Additional file 2: Table S1). The point estimates from each of the six high-rate treatments across all three methods had relatively narrow ranges in most cases (Additional file 3: Figure S2). However, RTT regression using TempEst produced rate estimates with a large spread when there was high rate variation among lineages. The Bayesian median estimates of rates had a small spread for sequence data that had been produced with a high rate, but these estimates tended to have an upward bias when there was high rate variation among lineages and high phylo-temporal clustering (Fig. 1; Additional file 2: Table S1).

**Fig. 2:**
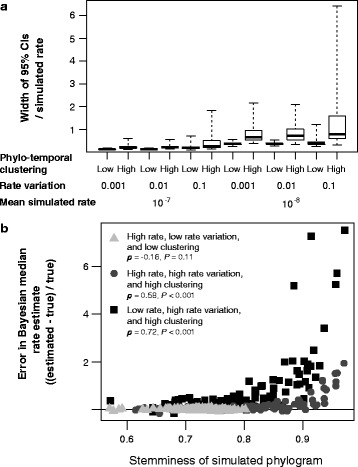
**a** Uncertainty in Bayesian estimates of substitution rates across 12 simulation conditions, as measured by the width of the 95% credibility interval of the estimate divided by the rate used for simulation. One hundred data sets were produced by simulation under distinct evolutionary conditions and analysed using BEAST. **b** Relationship between phylogenetic stemminess (proportion of overall tree length represented by internal branches) and the error in the Bayesian median rate estimates

For sequence data that have evolved with a low rate, RTT regression produced estimates that were accurate but had a large spread under all of the conditions examined (Fig. 1; Additional file 2: Table S2). Least-squares fitting systematically underestimated the rate but these estimates had a relatively small spread (Fig. 1), although not as small as seen in the estimates from data that had evolved with a high rate (Additional file 2: Table S3). The Bayesian rate estimates had a small spread when phylo-temporal clustering was low (Additional file 2: Table S4). However, this method produced overestimates of the rate, with the posterior medians often being more than 100% greater than the true rate, when low rate was combined with high phylo-temporal clustering (Fig. 2; Additional file 2: Table S1). RTT regression appeared to be the most robust to the interaction of these unfavourable factors (i.e. phylo-temporal clustering, low rate, and high rate variation among lineages), with the greatest similarity in outcomes across our simulation scenarios (Kruskal-Wallis χ^2^ = 13.7, d.f. = 11, *P* = 0.25). In contrast, rate estimates were different across simulation scenarios when obtained using LSD (Kruskal-Wallis χ^2^ = 357.21, d.f. = 11, *P* < 0.001) and BEAST (Kruskal-Wallis χ^2^ = 462.26, d.f. = 11, *P* < 0.001).

The data sets that yielded erroneous rate estimates when analysed using Bayesian inference tended to have phylograms in which internal branches represented a large proportion of the total tree length (high ‘stemminess’ [54]; Fig. 2; Additional file 4: Figure S3; Additional file 5: Figure S4; Additional file 6: Figure S5; Additional file 7: Figure S6). Since these trees have shorter terminal branches, the sum of their branch lengths is smaller than those of trees with low stemminess, leading to data sets with less information from which to estimate the rate. We found a positive correlation between phylogenetic stemminess and the spread of median posterior rate estimates in conditions of high rate variation and high clustering (*P* < 0.001; Additional file 7: Figure S6).

### Mitochondrial genomes

**Fig. 3:**
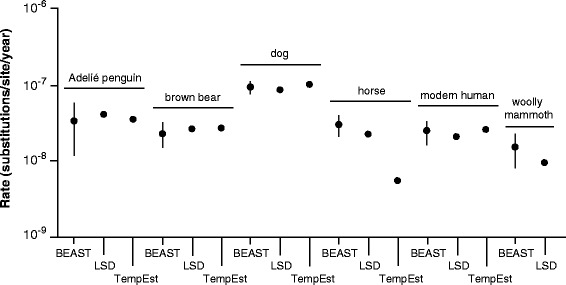
Estimates of substitution rates from time-structured mitogenomic data sets from six vertebrate species. Data were analysed using Bayesian inference in BEAST, least-squares dating in LSD, and regression of root-to-tip distances in TempEst. Bayesian estimates are indicated by their median and 95% credibility intervals. Regression of root-to-tip distances failed to yield a positive rate estimate from the mitogenomes from woolly mammoth, so no rate estimate is shown for TempEst. Details of the six data sets are given in Table 1

Our analyses of mitogenomic data sets from six vertebrate species produced rate estimates that were largely congruent with one another even when the data showed evidence of among-lineage rate variation (Table 2; Fig. 3). The horse mitogenomes presented an exception to this, with RTT regression producing a much lower rate estimate than the other two methods. The samples in this data set showed strong phylo-temporal clustering (*P* < 0.001), as did the dog mitogenome sequences. Almost all of the data sets passed both of the criteria considered for the date-randomization test for temporal structure, with the exception of the mitogenomes from the Adélie penguin (Table 2).

**Table 2:** Results from analyses of six time-structured mitogenomic data sets

Species	Clock model^a^	Tree prior^a^	Phylo-temporal clustering^b^ (*P*-value)	Date-randomization test^c^	
				CR1	CR2
Adélie penguin	Strict	Constant size	0.079	Fail	Fail
Brown/polar bear	Strict	Constant size	0.168	Pass	Pass
Dog	Relaxed	Constant size	0.006	Pass	Pass
Horse	Strict	Constant size	< 0.001	Pass	Pass
Modern human	Strict	Skyride	0.166	Pass	Pass
Woolly mammoth	Relaxed	Constant size	0.075	Pass	Pass

^a^Clock models and tree priors were compared using marginal likelihoods estimated by stepping-stone sampling. Marginal likelihoods are given in Additional file 2: Table S5

^b^*P*-values below 0.05 indicate that sequences with similar ages tend to be clustered together in the phylogenetic tree

^c^We considered two criteria that have been proposed for the date-randomization test, CR1 and CR2 [27]. These criteria are described in the Methods. Our results are based on 20 date-randomized replicates

Our rate estimates from the mitogenomes of Adélie penguin were 3.54 × 10^− 8^ subs/site/year (RTT regression), 4.10 × 10^− 8^ subs/site/year (least-squares dating), and 3.37 × 10^− 8^ subs/site/year (95% credibility interval 1.16–5.86 × 10^− 8^ subs/site/year; BEAST). These are mutually consistent but are higher than the Bayesian estimate of 1.8–2.4 × 10^− 8^ subs/site/year reported previously [23]. The original study assumed a relaxed clock, whereas we used a strict-clock model as selected by comparison of marginal likelihoods (Table 2, Additional file 2: Table S5). The discrepancy between rate estimates is potentially explained by the lack of temporal structure in the data set, as indicated by the failure of the data to meet either criterion of the date-randomization test (Table 2).

The results from our analyses of the mitogenomes from brown bears and polar bears are noteworthy because this data set contains a single ancient DNA sequence. The data appear to have temporal structure according to the date-randomization test (Table 2), confirming previous suggestions that a single ancient tip can be adequate for calibration provided that it is sufficiently old [32]. Our rate estimates are consistent with a previous estimate of 3.49 × 10^− 8^ subs/site/year from 95 samples of brown bears [55].

## Discussion

Our study demonstrates that three different methods are able to produce consistent estimates of substitution rates from ancient DNA data sets. In contrast with a previous study of virus data [31], we did not find that high among-lineage rate variation led to higher variability in rate estimates compared with our treatments involving low and medium rate variation. An exception to this is the Bayesian estimates from the sequence data from the simulations with a low substitution rate and high among-lineage variation, for which we recovered a much wider spread of median posterior estimates than for the other low-rate scenarios (Fig. 2). The discrepancy between our results and those from the previous study might be due to virus sequences having a considerably different mode and magnitude of among-lineage rate heterogeneity compared with the simulation conditions explored here.

We found that data sets with phylo-temporal clustering tended to yield more disparate rate estimates across the three methods compared here, a result that is consistent with that of a previous study of viruses [31]. This form of clustering might reduce the number of effective calibrations because it leads to fewer independent comparisons of genetic change and sampling times [28], resulting in increased uncertainty in the rate estimate. In Bayesian analyses, greater uncertainty can lead to substantial increases in the mean and median of the posterior distribution of the rate [9]. These patterns are seen in our rate estimates from the simulations with low substitution rates, for which least-squares fitting and Bayesian methods produced point estimates that had a greater bias and greater spread in the presence of pronounced phylo-temporal clustering. However, our interpretation of the Bayesian rate estimates here focus on the posterior medians as point values, whereas in practice the 95% credibility intervals should be taken into account.

Most of the mitogenome data sets showed some degree of phylo-temporal clustering, although this pattern was strongest in the sequences from dog and horse. In practice, phylo-temporal clustering is likely to be a prominent and unavoidable feature of ancient DNA data sets. This is because many data sets include samples from the same site or even the same stratum, and sampling is likely to be very uneven across geographic regions [34, 35]. Expanding the data set to include samples from multiple sites and multiple age layers will not always be feasible, owing to constraints on time, resources, and the availability of samples [34].

For each of the six mitogenome data sets, similar rate estimates were obtained from the three methods that we examined. The rate estimates for the horse mitogenomes were a notable exception to this pattern; strong phylo-temporal clustering in the data provides a possible explanation for the large discrepancy in the rate estimate from RTT regression. The lack of temporal signal in the mitogenomes from Adélie penguin is notable, because a mitochondrial D-loop data set from this species previously passed the date-randomization test despite having a much narrower sampling window [9, 56]. This result indicates that the spread of sampling times is not the sole determinant of temporal signal in time-structured data sets.

Our simulation study shows that different phylogenetic methods can produce congruent rate estimates if the substitution rate has been high and when there has been low to moderate rate variation among lineages. However, our results are consistent with those of previous studies in showing that the median posterior rates from Bayesian phylogenetic methods can be overestimates under certain conditions [9]. Rate estimates tended to have wider 95% credibility intervals when trees had high stemminess, a condition that is more likely when samples are drawn from a contracting population or when sequences are subject to purifying selection [57]. The increase in uncertainty reflects the lower information content in data sets that have evolved under these conditions. A potential solution is to widen the sampling window by including older sequences, although this might be difficult to achieve in practice [34]. We have not investigated the impacts of including a larger proportion of modern sequences, which can be done in studies of extant species.

The RTT regression method yielded mixed results in our simulation study, but it produced rate estimates comparable to those from least-squares fitting and Bayesian inference for four of the six mitogenomic data sets. Thus, despite its weaknesses, RTT regression can still be a useful qualitative complement to other methods because it can provide a rapid evaluation of the presence of a temporal signal in the data [11]. It also appears to be more robust to the confounding effects of low rate and phylo-temporal clustering. Sequence data that yield no apparent relationship between root-to-tip distance and sampling time should be further examined using more complex methods that allow rate variation among lineages.

The least-squares approach represents a valuable alternative to the more widely used methods of analysing ancient DNA sequences, which have been dominated by Bayesian methods [10]. As with RTT regression, least-squares fitting assumes a strict clock and attempts to fit data to a curve based on minimizing the statistical residual. Least-squares fitting does not aim to capture the evolutionary process that produces the sequence, but it is relatively robust to violations of the strict clock and can handle data with appreciable levels of among-lineage rate variation [13]. The method is particularly valuable for analyses of large data sets, for which the computational demands of a Bayesian phylogenetic analysis can be prohibitive [58].

## Conclusions

Our study has shown that three methods of rate estimation from time-structured data produce comparable estimates of substitution rates under various evolutionary conditions. These results are broadly consistent with those from analyses of time-structured sequence data from viruses [31] and from previous investigations of ancient DNA sequences [9, 22]. However, our analyses have provided new insights into how the three methods respond differently to the potentially confounding impacts of among-lineage rate variation and phylo-temporal clustering of sequences. This highlights the value of using all three methods to analyse ancient DNA data, and comparisons with the performance of other rate-estimation methods will be valuable [59]. Increasing the reliability of rate estimates will lead to a more accurate understanding of demographic and evolutionary processes on recent timescales.

## Additional files

Additional file 1: **Figure S1.** Simulations of sequence evolution were performed across 12 different scenarios, representing different combinations of **a** mean rate, **b** rate variation among lineages, and **c** phylo-temporal clustering. The conditions of the simulation scenarios are based on those observed in time-structured mitogenomic data sets. One hundred replicates were performed for each of the 12 scenarios. **d** Data were analysed using three different methods. (PDF 171 kb)

Additional file 2: **Table S1.** One-sample Wilcoxon tests of errors in rate estimates obtained using TempEst, LSD, and BEAST. Significant results are indicated in bold font. **Table S2.** Mann-Whitney-Wilcoxon pairwise comparisons between standardized errors in rate estimates obtained using TempEst. Rows and columns correspond to the 12 simulation scenarios. Significant results are indicated in bold font. **Table S3.** Mann-Whitney-Wilcoxon pairwise comparisons between standardized errors in rate estimates obtained using LSD. Rows and columns correspond to the 12 simulation scenarios. Significant results are indicated in bold font. **Table S4.** Mann-Whitney-Wilcoxon pairwise comparisons between standardized errors in rate estimates obtained using BEAST. Rows and columns correspond to the 12 simulation scenarios. Significant results are indicated in bold font. **Table S5.** Marginal likelihoods of different combinations of clock models and tree priors for six mitogenomic data sets. (DOCX 36 kb)

Additional file 3: **Figure S2.** Pairwise comparisons of rate estimates from regression of root-to-tip distances in TempEst, least-squares dating in LSD, and Bayesian inference in BEAST. Comparisons are between BEAST and TempEst (top), BEAST and LSD (middle), and TempEst and LSD ottom). The closer the fit of the points to the solid lines, the greater the congruence between the estimates from the two methods being compared. Dashed lines indicate a line of best fit for the estimates. The two distinct clouds of points within each panel represent the estimates from the data simulated with low and high rates. Proportional difference and bias were calculated as in a previous study by Duchêne et al. [31]. Proportional difference is the difference in the estimates between two methods, divided by the first rate estimate. Bias is the proportion of data sets for which the estimate along the x-axis is greater than that along the y-axis. (PDF 199 kb)

Additional file 4: **Figure S3.** Examples of phylogenetic trees with different degrees of stemminess, as measured by the proportion of the overall tree length represented by internal branches (values in parentheses). (PDF 40 kb)

Additional file 5: **Figure S4.** Relationships between phylogenetic stemminess and error in rate estimates using regression of root-to-tip distances in TempEst for 12 simulation treatments. Dashed lines indicate half an order of magnitude above or below the rates used for simulation. Light grey lines indicate lines of best fit for the estimates. (PDF 639 kb)

Additional file 6: **Figure S5.** Relationships between phylogenetic stemminess and error in rate estimates using least-squares dating in LSD for 12 simulation treatments. Dashed lines indicate half an order of magnitude above or below the rates used for simulation. Light grey lines indicate lines of best fit for the estimates. (PDF 634 kb)

Additional file 7: **Figure S6.** Relationships between phylogenetic stemminess and error in median posterior estimates using Bayesian inference in BEAST for 12 simulation treatments. Dashed lines indicate half an order of magnitude above or below the rates used for simulation. Light grey lines indicate lines of best fit for the estimates. (PDF 643 kb)

## Abbreviations

MCMC: Markov chain Monte Carlo
RTT: root-to-tip
