# Supplementary material for: A comparison of methods for estimating substitution rates from ancient DNA sequence data
Source: BMC Evol Biol. 2018 May 16;18:70. doi: 10.1186/s12862-018-1192-3 (PMC5956955; doi:10.1186/s12862-018-1192-3)
Supplement: Supplementary file 1 — Figure S1. Simulations of sequence evolution were performed across 12 different scenarios, representing different combinations of a mean rate, b rate variation among lineages, and c phylo-temporal clustering. The conditions of the simulation scenarios are based on those observed in time-structured mitogenomic data sets. One hundred replicates were performed for each of the 12 scenarios. d Data were analysed using three different methods. (PDF 171 kb) [file 12862_2018_1192_MOESM1_ESM.pdf]

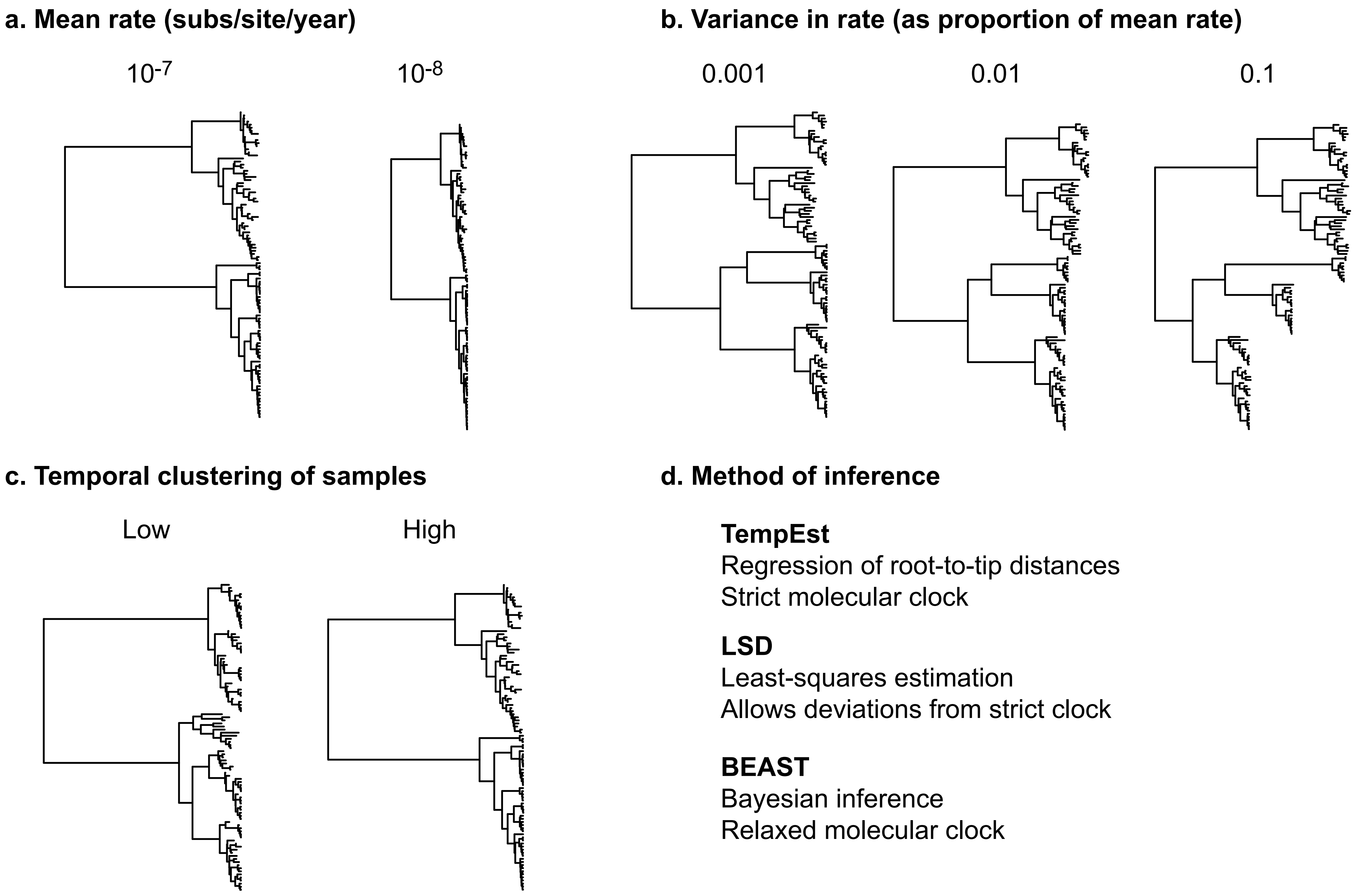
a. Mean rate (subs/site/year)
10-7
10-8
b. Variance in rate (as proportion of mean rate)
0.001
0.01
0.1
c. Temporal clustering of samples
Low
High
d. Method of inference
TempEst
Regression of root-to-tip distances
Strict molecular clock
LSD
Least-squares estimation
Allows deviations from strict clock
BEAST
Bayesian inference
Relaxed molecular clock
