# Supplementary material for: A comparison of methods for estimating substitution rates from ancient DNA sequence data
Source: BMC Evol Biol. 2018 May 16;18:70. doi: 10.1186/s12862-018-1192-3 (PMC5956955; doi:10.1186/s12862-018-1192-3)
Supplement: Supplementary file 2 — Table S1. One-sample Wilcoxon tests of errors in rate estimates obtained using TempEst, LSD, and BEAST. Significant results are indicated in bold font. Table S2. Mann-Whitney-Wilcoxon pairwise comparisons between standardized errors in rate estimates obtained using TempEst. Rows and columns correspond to the 12 simulation scenarios. Significant results are indicated in bold font. Table S3. Mann-Whitney-Wilcoxon pairwise comparisons between standardized errors in rate estimates obtained using LSD. Rows and columns correspond to the 12 simulation scenarios. Significant results are indicated in bold font. Table S4. Mann-Whitney-Wilcoxon pairwise comparisons between standardized errors in rate estimates obtained using BEAST. Rows and columns correspond to the 12 simulation scenarios. Significant results are indicated in bold font. Table S5. Marginal likelihoods of different combinations of clock models and tree priors for six mitogenomic data sets. (DOCX 36 kb) [file 12862_2018_1192_MOESM2_ESM.docx]

**Table S1.** One-sample Wilcoxon tests of errors in rate estimates obtained using TempEst, LSD, and BEAST. Significant results are indicated in bold font.

| **Mean simulated rate** (subs/site/year) | **10^-7^** | | | | | | **10^-8^** | | | | | |
| --- | --- | --- | --- | --- | --- | --- | --- | --- | --- | --- | --- | --- |
| **Rate variation** (% expected no. of substitutions) | **0.001** | | **0.01** | | **0.1** | | **0.001** | | **0.01** | | **0.1** | |
| **Phylo-temporal clustering** | **Low** | **High** | **Low** | **High** | **Low** | **High** | **Low** | **High** | **Low** | **High** | **Low** | **High** |
| **TempEst** | V=2163  *P*=0.214 | V=3036  *P*=0.079 | V=1967  *P*=0.055 | V=2323  *P*=0.488 | V=2482  *P*=0.884 | V=2859  *P*=0.252 | V=2407  *P*=0.686 | V=2712  *P*=0.521 | V=2513  *P*=0.968 | V=2513  *P*=0.968 | V=1921  *P*=0.038 | V=2953  *P*=0.142 |
| **LSD** | V=2278.5  *P*=0.398 | V=2813  *P*=0.323 | V=1996  *P*=0.069 | V=2224  *P*=0.301 | V=2438.5  *P*=0.9 | V=2236.5  *P*=0.504 | **V=671.5**  ***P*<0.001** | **V=569**  ***P*<0.001** | **V=406**  ***P*<0.001** | **V=227**  ***P*<0.001** | **V=221**  ***P*<0.001** | **V=520**  ***P*<0.001** |
| **BEAST** | V=2744  *P*=0.26 | **V=4184**  ***P*<0.001** | V=2129  *P*=0.374 | **V=4153**  ***P*<0.001** | **V=3965**  ***P*<0.001** | **V=4509**  ***P*<0.001** | **V=3450**  ***P*=0.001** | **V=4534**  ***P*<0.001** | V=2653  *P*=0.536 | **V=4419**  ***P*<0.001** | V=3091  *P*=0.032 | **V=4828**  ***P*<0.001** |

**Table S2.** Mann-Whitney-Wilcoxon pairwise comparisons between standardized errors in rate estimates obtained using TempEst. Rows and columns correspond to the 12 simulation scenarios. Significant results are indicated in bold font.

| **Mean simulated rate** (subs/site/year) | | | **10^-7^** | | | | | | **10^-8^** | | | | | |
| --- | --- | --- | --- | --- | --- | --- | --- | --- | --- | --- | --- | --- | --- | --- |
| **Rate variation** (% expected no. of substitutions) | | | **0.001** | | **0.01** | | **0.1** | | **0.001** | | **0.01** | | **0.1** | |
| **Phylo-temporal clustering** | | | **Low** | **High** | **Low** | **High** | **Low** | **High** | **Low** | **High** | **Low** | **High** | **Low** | **High** |
| **10^-7^** | **0.001** | **Low** | W=5000  *P*=1 | **W=4082**  ***P*=0.025** | W=5031.5  *P*=0.94 | W=4782  *P*=0.595 | W=4910.5  *P*=0.828 | W=4292  *P*=0.084 | **W=10000**  ***P*<0.001** | **W=10000**  ***P*<0.001** | **W=10000**  ***P*<0.001** | **W=10000**  ***P*<0.001** | **W=10000**  ***P*<0.001** | **W=10000**  ***P*<0.001** |
|  |  | **High** |  | W=5000  *P*=1 | **W=6029**  ***P*=0.012** | W=5760  *P*=0.063 | W=5435.5  *P*=0.288 | W=4969.5  *P*=0.942 | **W=10000**  ***P*<0.001** | **W=10000**  ***P*<0.001** | **W=10000**  ***P*<0.001** | **W=10000**  ***P*<0.001** | **W=10000**  ***P*<0.001** | **W=10000**  ***P*<0.001** |
|  | **0.01** | **Low** |  |  | W=5000  *P*=1 | W=4763  *P*=0.563 | W=4877  *P*=0.765 | **W=4157**  ***P*=0.04** | **W=10000**  ***P*<0.001** | **W=10000**  ***P*<0.001** | **W=10000**  ***P*<0.001** | **W=10000**  ***P*<0.001** | **W=10000**  ***P*<0.001** | **W=10000**  ***P*<0.001** |
|  |  | **High** |  |  |  | W=5000  *P*=1 | W=5013  *P*=0.976 | W=4381.5  *P*=0.131 | **W=10000**  ***P*<0.001** | **W=10000**  ***P*<0.001** | **W=10000**  ***P*<0.001** | **W=10000**  ***P*<0.001** | **W=10000**  ***P*<0.001** | **W=10000**  ***P*<0.001** |
|  | **0.1** | **Low** |  |  |  |  | W=5000  *P*=1 | W=4703  *P*=0.469 | **W=9484**  ***P*<0.001** | **W=9478**  ***P*<0.001** | **W=9491**  ***P*<0.001** | **W=9482**  ***P*<0.001** | **W=9491**  ***P*<0.001** | **W=9479**  ***P*<0.001** |
|  |  | **High** |  |  |  |  |  | W=5000  *P*=1 | **W=10000**  ***P*<0.001** | **W=10000**  ***P*<0.001** | **W=10000**  ***P*<0.001** | **W=10000**  ***P*<0.001** | **W=10000**  ***P*<0.001** | **W=10000**  ***P*<0.001** |
| **10^-8^** | **0.001** | **Low** |  |  |  |  |  |  | W=5000  *P*=1 | W=4705  *P*=0.472 | W=4888  *P*=0.785 | W=4927  *P*=0.859 | W=5579.5  *P*=0.157 | W=4452  *P*=0.181 |
|  |  | **High** |  |  |  |  |  |  |  | W=5000  *P*=1 | W=5238  *P*=0.562 | W=5222  *P*=0.588 | **W=5833.5**  ***P*=0.042** | W=4744  *P*=0.532 |
|  | **0.01** | **Low** |  |  |  |  |  |  |  |  | W=5000  *P*=1 | W=4918  *P*=0.842 | W=5662  *P*=0.106 | W=4523  *P*=0.244 |
|  |  | **High** |  |  |  |  |  |  |  |  |  | W=5000  *P*=1 | W=5646  *P*=0.115 | W=4556  *P*=0.279 |
|  | **0.1** | **Low** |  |  |  |  |  |  |  |  |  |  | W=5000  *P*=1 | **W=3944.5**  ***P*=0.01** |
|  |  | **High** |  |  |  |  |  |  |  |  |  |  |  | W=5000  *P*=1 |

**Table S3.** Mann-Whitney-Wilcoxon pairwise comparisons between standardized errors in rate estimates obtained using LSD. Rows and columns correspond to the 12 simulation scenarios. Significant results are indicated in bold font.

| **Mean simulated rate** (subs/site/year) | | | **10^-7^** | | | | | | **10^-8^** | | | | | |
| --- | --- | --- | --- | --- | --- | --- | --- | --- | --- | --- | --- | --- | --- | --- |
| **Rate variation** (% expected no. of substitutions) | | | **0.001** | | **0.01** | | **0.1** | | **0.001** | | **0.01** | | **0.1** | |
| **Phylo-temporal clustering** | | | **Low** | **High** | **Low** | **High** | **Low** | **High** | **Low** | **High** | **Low** | **High** | **Low** | **High** |
| **10^-7^** | **0.001** | **Low** | W=5000  *P=*1 | W=4441  *P*=0.172 | W=5305.5  *P*=0.456 | W=5254.5  *P*=0.535 | W=4878.5  *P*=0.767 | W=5161.5  *P*=0.694 | **W=10000**  ***P*<0.001** | **W=10000**  ***P*<0.001** | **W=10000**  ***P*<0.001** | **W=10000**  ***P*<0.001** | **W=10000**  ***P*<0.001** | **W=10000**  ***P*<0.001** |
|  |  | **High** |  | W=5000  *P*=1 | **W=5853**  ***P*=0.037** | W=5695  *P*=0.09 | W=5230  *P*=0.575 | W=5545  *P*=0.183 | **W=10000**  ***P*<0.001** | **W=10000**  ***P*<0.001** | **W=10000**  ***P*<0.001** | **W=10000**  ***P*<0.001** | **W=10000**  ***P*<0.001** | **W=10000**  ***P*<0.001** |
|  | **0.01** | **Low** |  |  | W=5000  *P*=1 | W=4976  *P*=0.954 | W=4656.5  *P*=0.402 | W=4899  *P*=0.806 | **W=10000**  ***P*<0.001** | **W=10000**  ***P*<0.001** | **W=10000**  ***P*<0.001** | **W=10000**  ***P*<0.001** | **W=10000**  ***P*<0.001** | **W=10000**  ***P*<0.001** |
|  |  | **High** |  |  |  | W=5000  *P*=1 | W=4831  *P*=0.681 | W=4964  *P*=0.931 | **W=10000**  ***P*<0.001** | **W=10000**  ***P*<0.001** | **W=10000**  ***P*<0.001** | **W=10000**  ***P*<0.001** | **W=10000**  ***P*<0.001** | **W=10000**  ***P*<0.001** |
|  | **0.1** | **Low** |  |  |  |  | W=5000  *P*=1 | W=5118.5  *P*=0.773 | **W=10000**  ***P*<0.001** | **W=10000**  ***P*<0.001** | **W=10000**  ***P*<0.001** | **W=10000**  ***P*<0.001** | **W=10000**  ***P*<0.001** | **W=10000**  ***P*<0.001** |
|  |  | **High** |  |  |  |  |  | W=5000  *P*=1 | **W=10000**  ***P*<0.001** | **W=10000**  ***P*<0.001** | **W=10000**  ***P*<0.001** | **W=10000**  ***P*<0.001** | **W=10000**  ***P*<0.001** | **W=10000**  ***P*<0.001** |
| **10^-8^** | **0.001** | **Low** |  |  |  |  |  |  | W=5000  *P*=1 | **W=6252.5**  ***P*=0.002** | W=5464  *P*=0.257 | **W=6632.5**  ***P*<0.001** | **W=6072**  ***P*=0.009** | **W=6048.5**  ***P*=0.01** |
|  |  | **High** |  |  |  |  |  |  |  | W=5000  *P*=1 | **W=4142**  ***P*=0.036** | W=5238.5  *P*=0.561 | W=4609.5  *P*=0.341 | W=4787  *P*=0.604 |
|  | **0.01** | **Low** |  |  |  |  |  |  |  |  | W=5000  *P*=1 | W=6206.5  *P*=0.003 | W=5587.5  *P*=0.151 | W=5595  *P*=0.146 |
|  |  | **High** |  |  |  |  |  |  |  |  |  | W=5000  *P*=1 | W=4329.5  *P*=0.102 | W=4514.5  *P*=0.236 |
|  | **0.1** | **Low** |  |  |  |  |  |  |  |  |  |  | W=5000  *P*=1 | W=5116  *P*=0.778 |
|  |  | **High** |  |  |  |  |  |  |  |  |  |  |  | W=5000  *P*=1 |

**Table S4.** Mann-Whitney-Wilcoxon pairwise comparisons between standardized errors in mean rate estimates obtained using BEAST. Rows and columns correspond to the 12 simulation scenarios. Significant results are indicated in bold font.

| **Mean simulated rate** (subs/site/year) | | | **10^-7^** | | | | | | **10^-8^** | | | | | |
| --- | --- | --- | --- | --- | --- | --- | --- | --- | --- | --- | --- | --- | --- | --- |
| **Rate variation** (% expected no. of substitutions) | | | **0.001** | | **0.01** | | **0.1** | | **0.001** | | **0.01** | | **0.1** | |
| **Phylo-temporal clustering** | | | **Low** | **High** | **Low** | **High** | **Low** | **High** | **Low** | **High** | **Low** | **High** | **Low** | **High** |
| **10^-7^** | **0.001** | **Low** | W=4802  *P*=1 | **W=2790**  ***P*<0.001** | W=5333  *P*=0.141 | **W=3152**  ***P*<0.001** | **W=3122**  ***P*<0.001** | **W=1537**  ***P*<0.001** | **W=9800**  ***P*<0.001** | **W=9408**  ***P*<0.001** | **W=9702**  ***P*<0.001** | **W=9212**  ***P*<0.001** | **W=9702**  ***P*<0.001** | **W=9604**  ***P*<0.001** |
|  |  | **High** |  | W=4802  *P*=1 | **W=7097**  ***P*<0.001** | W=5062  *P*=0.689 | W=4985  *P*=0.646 | **W=2710**  ***P*<0.001** | **W=9800**  ***P*<0.001** | **W=9408**  ***P*<0.001** | **W=9702**  ***P*<0.001** | **W=9212**  ***P*<0.001** | **W=9702**  ***P*<0.001** | **W=9604**  ***P*<0.001** |
|  | **0.01** | **Low** |  |  | W=4704.5  *P*=1 | **W=2733**  ***P*<0.001** | **W=2691**  ***P*<0.001** | **W=1305**  ***P*<0.001** | **W=9700**  ***P*<0.001** | **W=9312**  ***P*<0.001** | **W=9603**  ***P*<0.001** | **W=9118**  ***P*<0.001** | **W=9603**  ***P*<0.001** | **W=9506**  ***P*<0.001** |
|  |  | **High** |  |  |  | W=5000  *P*=1 | W=4942  *P*=0.918 | **W=2697**  ***P*<0.001** | **W=10000**  ***P*<0.001** | **W=9600**  ***P*<0.001** | **W=9900**  ***P*<0.001** | **W=9400**  ***P*<0.001** | **W=9900**  ***P*<0.001** | **W=9800**  ***P*<0.001** |
|  | **0.1** | **Low** |  |  |  |  | W=4802  *P*=1 | **W=2699**  ***P*<0.001** | **W=9800**  ***P*<0.001** | **W=9408**  ***P*<0.001** | **W=9702**  ***P*<0.001** | **W=9212**  ***P*<0.001** | **W=9702**  ***P*<0.001** | **W=9604**  ***P*<0.001** |
|  |  | **High** |  |  |  |  |  | W=4704.5  *P*=1 | **W=9700**  ***P*<0.001** | **W=9312**  ***P*<0.001** | **W=9603**  ***P*<0.001** | **W=9118**  ***P*<0.001** | **W=9603**  ***P*<0.001** | **W=9505**  ***P*<0.001** |
| **10^-8^** | **0.001** | **Low** |  |  |  |  |  |  | W=5000  *P*=1 | **W=1804**  ***P*<0.001** | W=5719  *P*=0.059 | **W=1236**  ***P*<0.001** | W=5174  P=0.582 | **W=791**  ***P*<0.001** |
|  |  | **High** |  |  |  |  |  |  |  | W=4608  *P*=1 | **W=8159**  ***P*<0.001** | W=4082  *P*=0.257 | **W=7728**  ***P*<0.001** | **W=2995**  ***P*<0.001** |
|  | **0.01** | **Low** |  |  |  |  |  |  |  |  | W=4900.5  *P*=1 | **W=868**  ***P*<0.001** | W=4396  P=0.211 | **W=545**  ***P*<0.001** |
|  |  | **High** |  |  |  |  |  |  |  |  |  | W=4418  *P*=1 | **W=8033**  ***P*<0.001** | **W=3190**  ***P*<0.001** |
|  | **0.1** | **Low** |  |  |  |  |  |  |  |  |  |  | W=4900.5  P=1 | **W=849**  ***P*<0.001** |
|  |  | **High** |  |  |  |  |  |  |  |  |  |  |  | W=4802  P=1 |

**Table S5.** Marginal likelihoods of different combinations of clock models and tree priors for six mitogenomic data sets. Best models are shown in bold font. Simpler models were preferred over more complex models if they were within 1 log likelihood unit.

|  | **Log marginal likelihood** | | | |
| --- | --- | --- | --- | --- |
|  | **Strict clock** | | **Uncorrelated lognormal relaxed clock** | |
|  | **Constant-size coalescent** | **Skyride coalescent** | **Constant-size coalescent** | **Skyride coalescent** |
| Adélie penguin | **-19943.0** | -19947.4 | -19942.4 | -19946.6 |
| Brown/polar bear | **-21130.8** | -21148.1 | -21130.6 | -21138.2 |
| Dog | -29807.4 | -29873.8 | **-29781.6** | -29831.0 |
| Horse | **-32164.0** | -32256.6 | -32163.3 | -32194.9 |
| Modern human | -26356.2 | **-26169.0** | -26351.1 | -26171.9 |
| Woolly mammoth | -26165.6 | -26191.8 | **-26150.4** | -26165.5 |
