# Supplementary figures and images for: A comparison of methods for estimating substitution rates from ancient DNA sequence data
Source: BMC Evol Biol. 2018 May 16;18:70. doi: 10.1186/s12862-018-1192-3 (PMC5956955; doi:10.1186/s12862-018-1192-3)

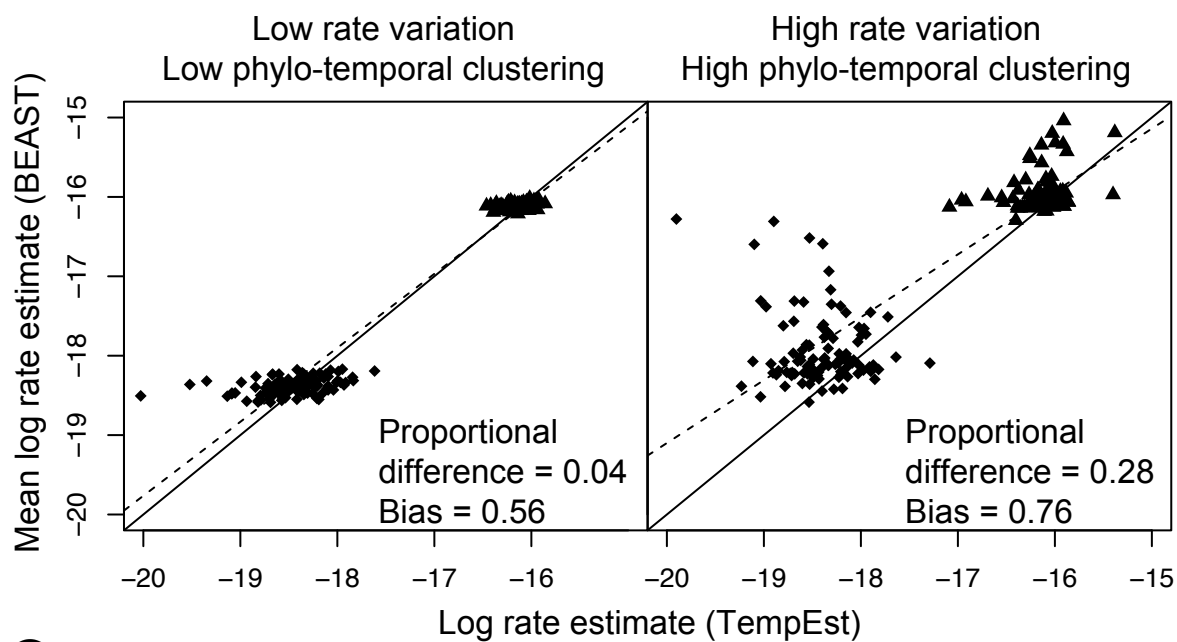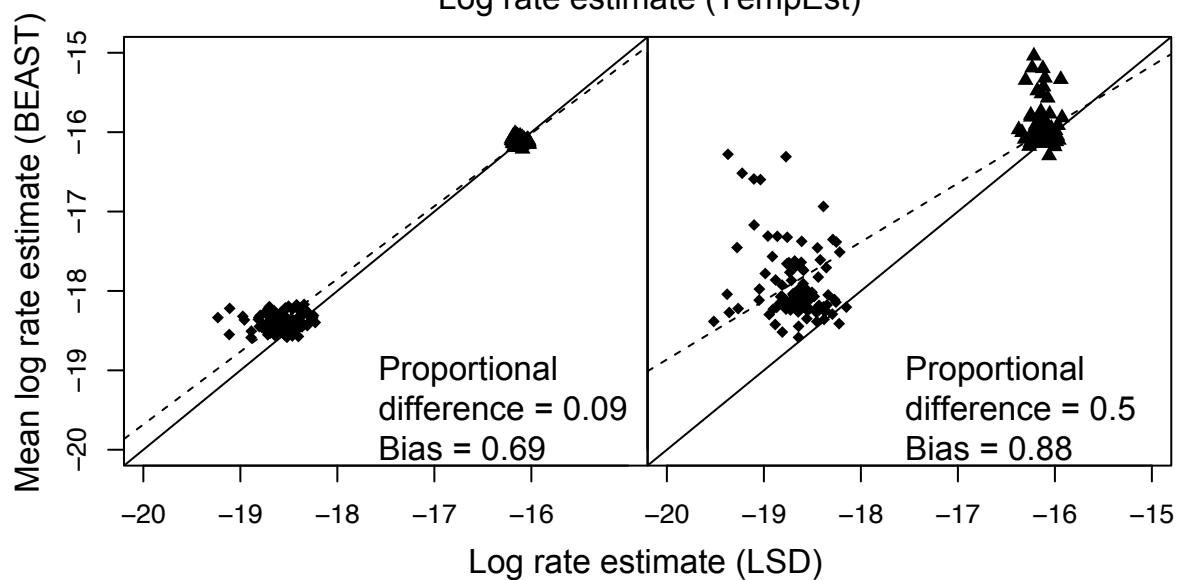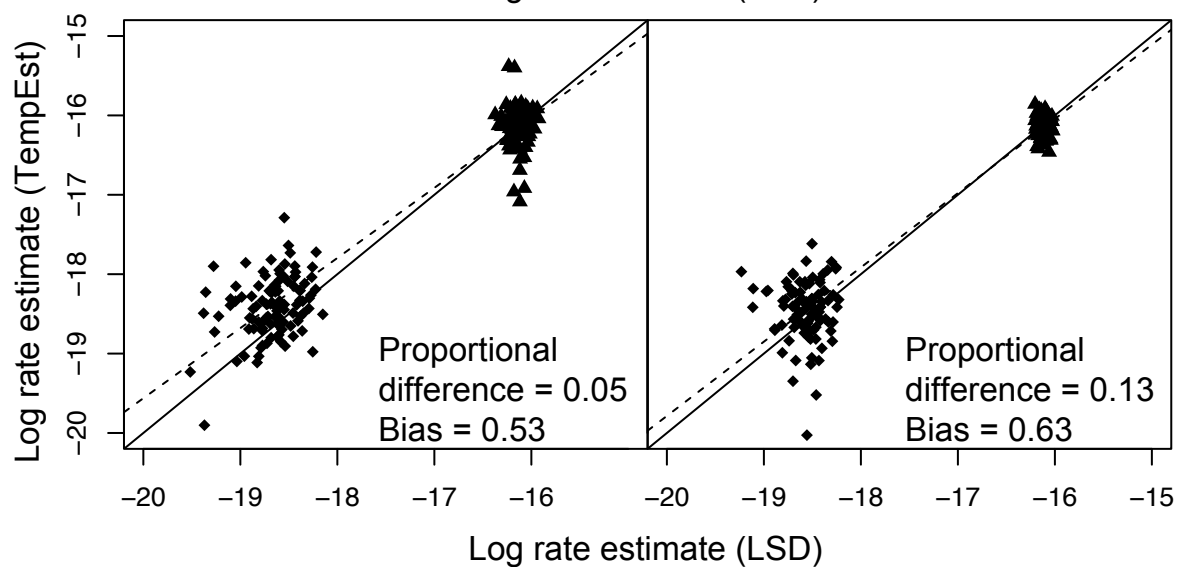

Supplement: Supplementary file 3 — Figure S2. Pairwise comparisons of rate estimates from regression of root-to-tip distances in TempEst, least-squares dating in LSD, and Bayesian inference in BEAST. Comparisons are between BEAST and TempEst (top), BEAST and LSD (middle), and TempEst and LSD ottom). The closer the fit of the points to the solid lines, the greater the congruence between the estimates from the two methods being compared. Dashed lines indicate a line of best fit for the estimates. The two distinct clouds of points within each panel represent the estimates from the data simulated with low and high rates. Proportional difference and bias were calculated as in a previous study by Duchêne et al. [31]. Proportional difference is the difference in the estimates between two methods, divided by the first rate estimate. Bias is the proportion of data sets for which the estimate along the x-axis is greater than that along the y-axis. (PDF 199 kb) [file 12862_2018_1192_MOESM3_ESM.pdf]

Low stemminess  
(0.51)

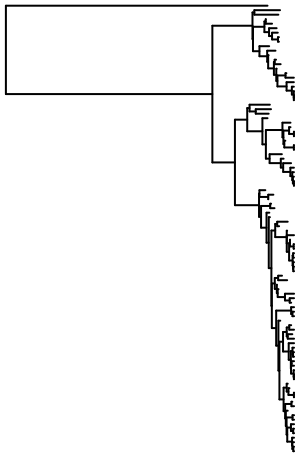

High stemminess  
(0.86)

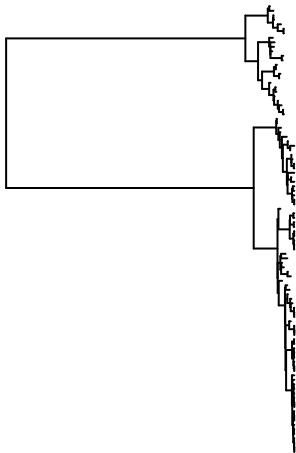

Supplement: Supplementary file 4 — Figure S3. Examples of phylogenetic trees with different degrees of stemminess, as measured by the proportion of the overall tree length represented by internal branches (values in parentheses). (PDF 40 kb) [file 12862_2018_1192_MOESM4_ESM.pdf]

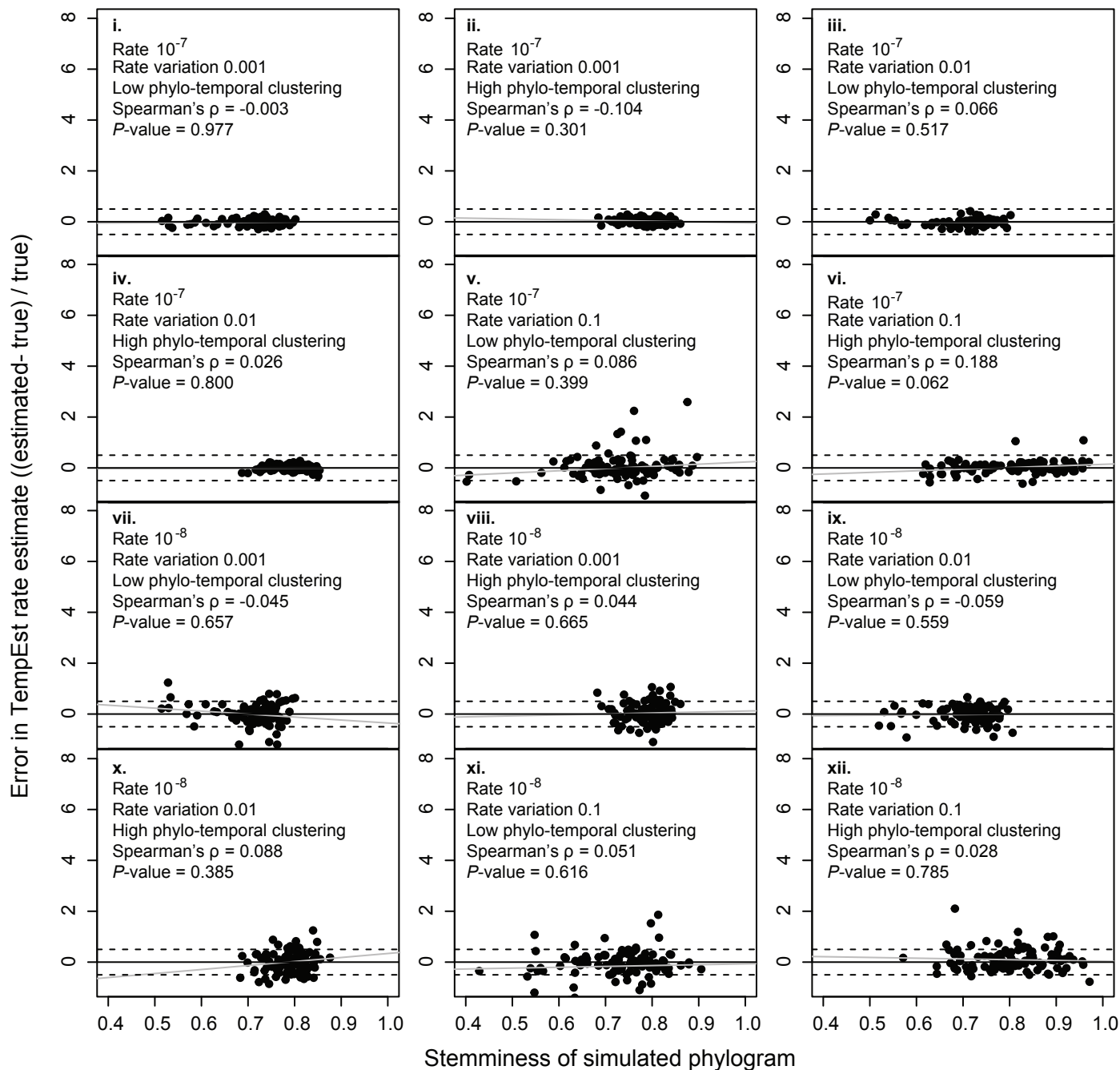

Supplement: Supplementary file 5 — Figure S4. Relationships between phylogenetic stemminess and error in rate estimates using regression of root-to-tip distances in TempEst for 12 simulation treatments. Dashed lines indicate half an order of magnitude above or below the rates used for simulation. Light grey lines indicate lines of best fit for the estimates. (PDF 639 kb) [file 12862_2018_1192_MOESM5_ESM.pdf]

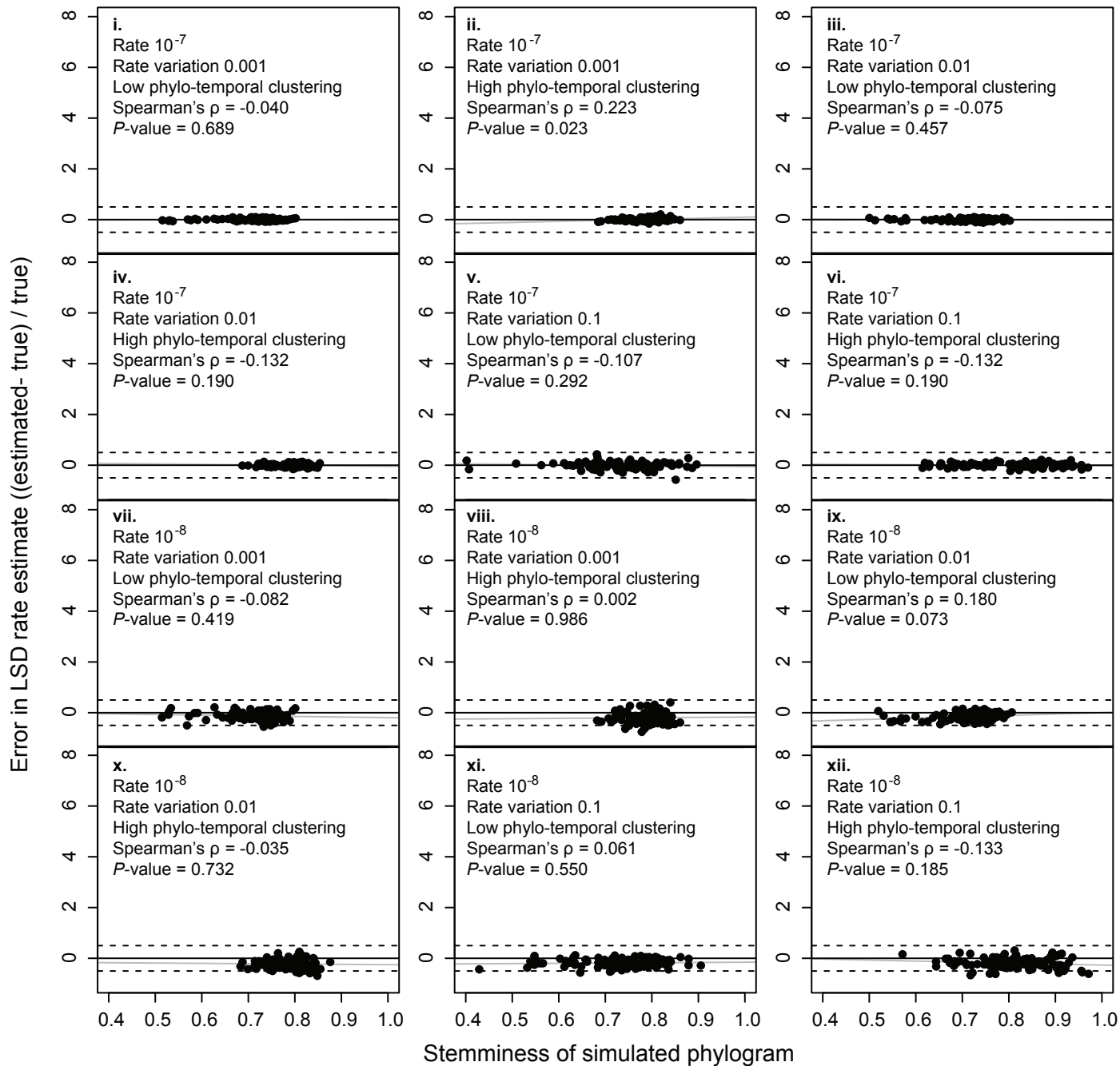

Supplement: Supplementary file 6 — Figure S5. Relationships between phylogenetic stemminess and error in rate estimates using least-squares dating in LSD for 12 simulation treatments. Dashed lines indicate half an order of magnitude above or below the rates used for simulation. Light grey lines indicate lines of best fit for the estimates. (PDF 634 kb) [file 12862_2018_1192_MOESM6_ESM.pdf]

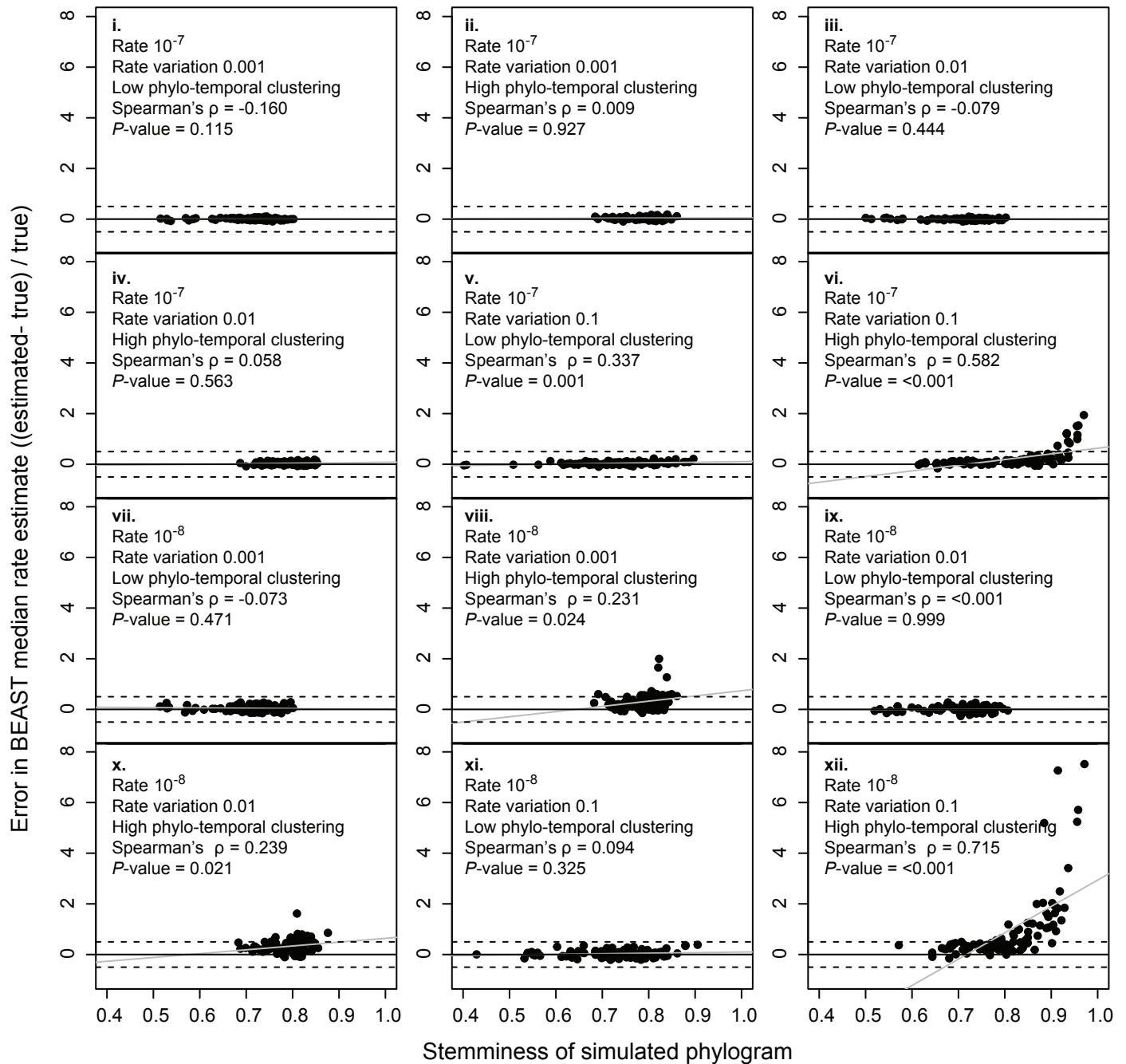

Supplement: Supplementary file 7 — Figure S6. Relationships between phylogenetic stemminess and error in median posterior estimates using Bayesian inference in BEAST for 12 simulation treatments. Dashed lines indicate half an order of magnitude above or below the rates used for simulation. Light grey lines indicate lines of best fit for the estimates. (PDF 643 kb) [file 12862_2018_1192_MOESM7_ESM.pdf]
